# Takayasu's Arteritis: An Uncommon Cause of Hemorrhagic Stroke in Young Individuals

**DOI:** 10.7759/cureus.52301

**Published:** 2024-01-15

**Authors:** Ebad-Ur Rehman Syed, Noman Salih, Hidayat Ullah, Abdul Wahab, Muhammad Younas Ali, Muhammad Ayub, Numan Ghani

**Affiliations:** 1 Medicine, Royal College of Surgeons in Ireland, Busaiteen, BHR; 2 Internal Medicine, Hayatabad Medical Complex, Peshawar, PAK; 3 General Practice, Hayatabad Medical Complex, Peshawar, PAK; 4 Medicine and Surgery, Gajju Khan Medical College, Swabi, PAK; 5 General Internal Medicine (GIM), University Hospital Wishaw, Wishaw, GBR; 6 Internal Medicine, Lady Reading Hospital Medical Teaching Institute (MTI), Peshawar, PAK

**Keywords:** asian woman, takayasu's arteritis, ischemic infarcts, prednisolone, aorta, vasculitis, neurovascular complications, cyclophosphamide, seizures

## Abstract

The aorta is particularly damaged by Takayasu's arteritis (TA), a rare form of vasculitis. Chest discomfort, exhaustion, fever, elevated blood pressure, heart failure, and stroke can all result from this. Major intimal fibrosis with vascular constriction is the disease's hallmark; although anybody can have it, Asian females in their 20s or 30s seem to be most typically affected. The treatment of a 23-year-old Asian female with Takayasu's arteritis (TA) is discussed in this case study, along with her presentation. Before developing seizures, the patient first showed signs of left-sided weakness and facial droop. Ischemic infarcts and vasculopathy were detected by imaging. The patient fulfilled several American College of Rheumatology (ACR) criteria for TA with a positive erythrocyte sedimentation rate (ESR). During treatment, high-dose prednisolone, cyclophosphamide, and neuroprotective measures were used. The patient's attentiveness and mobility improved despite early complications, such as vascular friability. This case illustrates the difficulties and effective treatment of neurovascular problems connected to TA.

## Introduction

Takayasu's arteritis (TA) is a chronic disease affecting the pulmonary artery and the aorta and its major branches [1,2]. It is noteworthy that females in their 20s and 30s are more likely to experience it, and Asian nations, especially Japan, have the highest frequency of cases [3,4]. In sharp contrast to Japan, where there are 40 cases per million annually, TA is notably uncommon in the United States and Asian countries other than Japan, with an annual incidence of only 2.6 cases per million [5].

Even though the exact origin of TA is still unknown, current research indicates that it may be due to an immune system-triggered inflammatory process that ultimately results in vessel wall fibrosis [6]. Strokes become much more likely as a result of the substantial stenosis of the arteries caused by this fibrosis.

There are two primary stages to TA. General symptoms including fever, myalgia, arthralgia, tiredness, and weight loss are characteristic of the first systemic phase. The "pulseless phase" that follows is characterized by specific characteristics including hypertension, claudication, vascular stenosis, and a range of neurological symptoms such as headaches, dizziness, blurred vision, transient ischemic episodes (TIAs), and, most significantly, strokes [7].

According to statistics, 10%-20% of individuals with TA may have ischemic strokes [1,8,9], while there are no reported cases of hemorrhagic strokes in TA patients. It is interesting to note that although strokes are a known complication, TA seldom manifests itself as a stroke at first [10]. This particular feature highlights how crucial it is to comprehend and identify the wider range of symptoms connected to TA, since doing so may facilitate prompt diagnosis and treatment and avert major consequences such as strokes.

## Case presentation

A 23-year-old Asian female who had no notable medical history was brought to our emergency room after experiencing a one-day bout of unconsciousness and an abrupt onset of left-side weakness with drooping of the left face. She had faint peripheral pulses on the left side, nonexistent radial pulses on the right side of the upper limbs, and absent pulses in the left dorsalis pedis upon examination. She scored 6/15 on the Glasgow Coma Scale. Blood pressure differences were observed, with the left upper extremity measuring 95/60 mm Hg, the right lower extremity measuring 120/70 mm Hg, and the left lower extremity measuring 140/80 mm Hg. The right upper extremity had no recorded blood pressure. There were two carotid bruits found: a left and a right. A positive left Babinski sign, reduced left sidetone, and early flaccid paralysis on the left side were all seen during the neurological examination. Her right facial palsy was mild involving just the lower half of the face while sparing the right forehead. The patient had no known family history of rheumatologic, cardiac, or cerebrovascular illness, nor was she taking any drugs.

**Figure 1:**
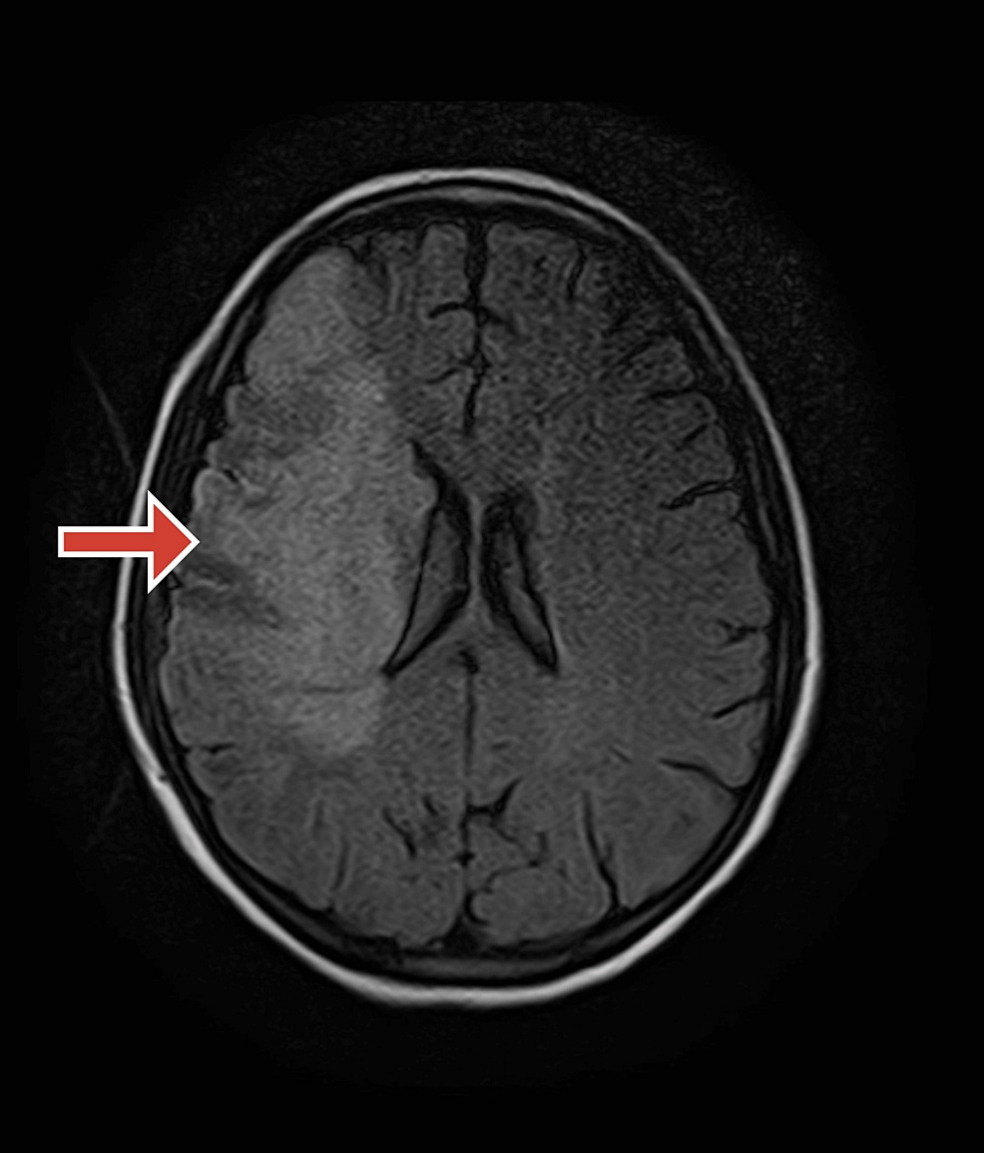
T1 MRI image with an arrow pointing to hemorrhagic infarct in the territory of the middle cerebral artery (MCA)

After 24 hours, a follow-up CT scan that was originally negative showed early indications of right parietal region ischemia. Since it was beyond the window period, antiplatelets were given instead of thrombolysis. Her symptoms grew worse after that, eventually resulting in seizures. Acute infarcts were indicated by aberrant hypointense regions in the right frontoparietal lobe and basal ganglia on a brain MRI with contrast. On T1 hyperintensity, hemorrhagic change was seen as shown in Figure 1.

**Figure 2:**
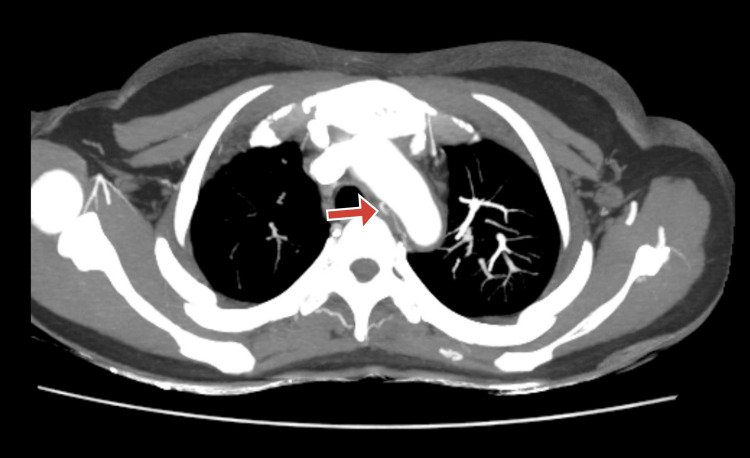
CT aortogram with an arrow showing the thickening of the wall of the arch of the aorta

**Figure 3:**
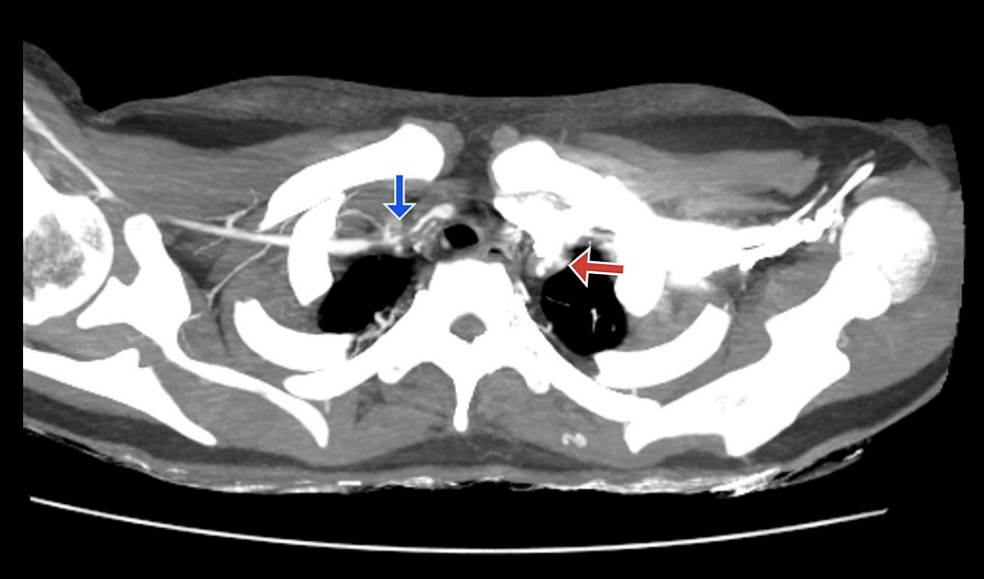
CT aortogram with the blue arrow showing the narrowing of the brachiocephalic trunk and the red arrow showing the narrowing of the left subclavian artery

**Figure 4:**
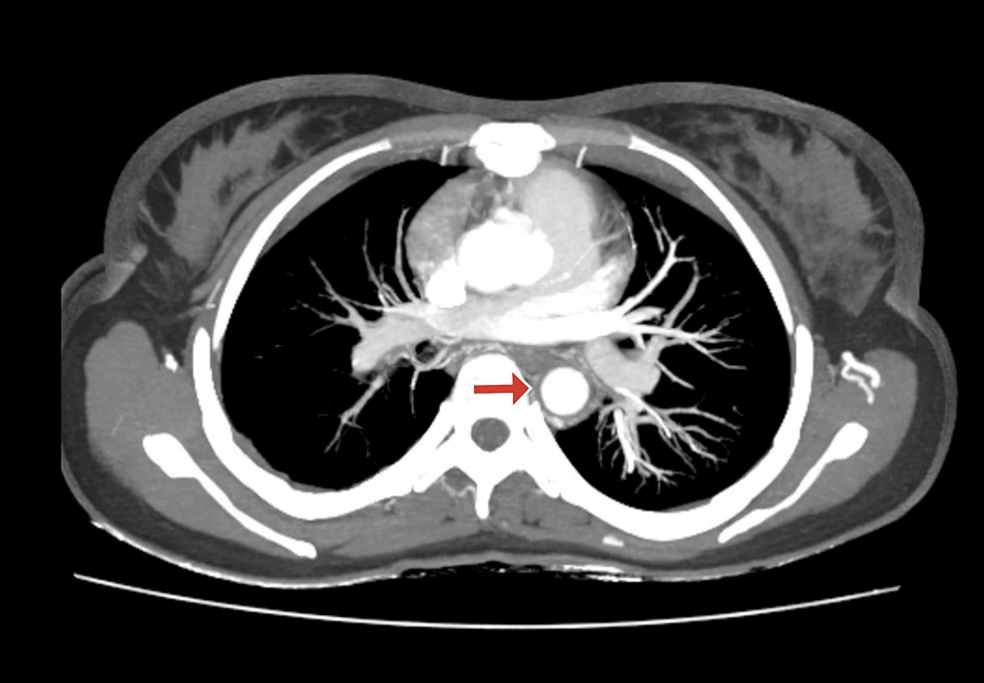
CT aortogram with the red arrow indicating the circumferential thickening of the wall of the descending aorta

A CT angiography (CTA) of the brain and carotid arteries was conducted due to suspicions of vascular anomalies. The results showed significant stenosis of the left common carotid artery, the brachiocephalic artery, the right common carotid artery, and the distal two-thirds of the right subclavian artery. Vasculitis and its aftereffects were verified by further imaging. To evaluate possible major arterial involvement, further imaging was performed. This included an ultrasound evaluation of the renal artery and all four limbs, which was unremarkable, and a CT aortogram, which is shown in Figures 2-4. Similar to CT angiography, the CT aortogram showed significant stenosis in the left common carotid artery, the right common carotid artery, the brachiocephalic artery, and the distal two-thirds of the right subclavian artery. The results of the CT angiography, the high erythrocyte sedimentation rate (ESR), and the clinical examination led to the tentative diagnosis of Takayasu's arteritis (TA). The patient satisfied more than three of the American College of Rheumatology's (ACR) 1990 TA criteria.

Blood pressure control, electrolyte balance, volume status management, and temperature modulation were among the neuroprotective techniques used. To lower intracranial pressure, mannitol was given at a dosage of 1 g/kg. In addition to steroids to lessen inflammation, the patient was given a tablet of nimodipine 50 mg four times daily (QID) for 21 days to regulate blood pressure and avoid vasoconstriction. Because of the vessel's friability in Takayasu's arteritis, surgical intervention was not sought despite consulting with neuro-interventional radiology.

The patient received prednisolone through an oral route at a rate of 1 mg/kg/day for the first five days, followed by a lower dose. Cyclophosphamide was administered through an intravenous (IV) route at a rate of 10 mg/kg every two weeks for three doses and then three weekly for three months starting from the 14th day of admission. The patient showed gradual improvement in her movement and alertness over the following weeks, more so on the left side. After staying in the hospital for three weeks, she was discharged with a daily dose of 60 mg of prednisolone and a recommendation for nursing care. The patient keeps visiting outpatient departments for neurology, neurosurgery, and ophthalmology. Occupational, physical, and speech therapies are all part of ongoing treatment, along with cyclophosphamide infusions and a maintenance dosage of 20 mg of prednisolone each day.

## Discussion

Unknown in origin, Takayasu's arteritis (TA) is a chronic inflammatory arteriopathy marked by thrombus development and stenotic alterations in the artery lumina [11]. It is predicted that between 10% and 20% of TA patients may experience an ischemic stroke, highlighting the seriousness of this illness and the requirement for increased clinical vigilance [1,8,9]; however, no data is available regarding hemorrhagic strokes in TA patients. Cerebrovascular problems can have a major influence on morbidity and mortality in persons with TA, and physicians should not ignore this possibility [12].

The development of a systematic approach targeted at reducing morbid events and untimely deaths is crucial in addressing the issues presented by stroke in patients with TA. It is critical to identify the nonspecific symptoms of TA, especially in younger stroke patients. These symptoms include fever, lethargy, dizziness, irregular pulse, and blood pressure fluctuations. As our patient's case demonstrated, prompt diagnosis is critical. Before being admitted to the hospital, the patient experienced arthralgia, low-grade fever, malaise, dizziness, and night sweats. Regretfully, the patient had a stroke before the diagnosis could be made. Prominent observations, such as extreme stenosis on imaging, irregular pulses, and variations in blood pressure, sparked concerns about TA and prompted the start of a rheumatologic examination.

In conclusion, stroke is an uncommon way for TA to manifest itself initially [10]. It becomes crucial to take TA into account when diagnosing stroke patients, particularly when there are no traditional risk factors present but dyslipidemia and/or hypertension [1,13]. It is also crucial to investigate the unique pathophysiology of stroke in TA, which calls for deeper research into the processes behind this event [14]. Systemic steroids are the first line of care for immunosuppression in TA, which is the aim of the treatment, although they are frequently linked to side effects and have a high recurrence rate [15]. As a result, for long-term illness management, nonsteroidal immunosuppressive medications such as methotrexate and cyclophosphamide are typically needed [15]. When applied comprehensively, this strategy can improve outcomes for patients with TA who are having cerebrovascular problems by improving early diagnosis and treatment.

## Conclusions

The care and presentation of a 23-year-old Asian female with Takayasu's arteritis (TA) are detailed in this case study. The patient began with drooping of the face and weakness on the left side, which developed into seizures. Vasculopathy and ischemic infarcts were detected by imaging. The patient satisfied several American College of Rheumatology (ACR) criteria for TA with a positive ESR. Cyclophosphamide, high-dose prednisolone, and neuroprotective strategies were all part of the treatment. The patient showed progress in awareness and mobility despite early complications, such as vascular friability. After three weeks, the patient was released from the hospital and is now receiving outpatient treatment, which includes rehabilitative therapy and a prescription for cyclophosphamide and prednisone.
